# Monozygotic multiple gestation following in vitro fertilization: analysis of seven cases from Japan

**DOI:** 10.1186/1743-1050-4-4

**Published:** 2007-09-22

**Authors:** Atsushi Yanaihara, Takeshi Yorimitsu, Hiroshi Motoyama, Hideaki Watanabe, Toshihiro Kawamura

**Affiliations:** 1 Denentoshi Ladies Clinic Reproductive Center, 2-3-10-5F Aoba-ku, Yokohama-shi, Kanagawa 227-0062, Japan; 2 Department of Obstetrics and Gynecology, Showa University School of Medicine, 1-5-8 Hatanodai, Shinagawa-ku, Tokyo 142-8666, Japan

## Abstract

We present a series of monozygous multiple gestations achieved following in vitro fertilization (IVF): one case of monochorionic triplet pregnancy and six cases of dizygotic triplet pregnancy. From September 2000 to December 2006, all patients achieving clinical pregnancy by ART were reviewed (*n *= 2433). A 37 year-old woman who delivered a healthy singleton after IVF returned two years later for FET, and a single blastocyst was transferred. This also resulted in pregnancy, but TV-USG revealed a single gestational sac with three distinct amniotic sacs, each containing a distinct fetal pole with cardiac activity. This pregnancy was electively terminated at nine weeks' gestation. An additional six cases of dizygotic triplets established after fresh embryo transfer (no ICSI or assisted hatching) are also described. Of these, one resulted in a miscarriage at eight weeks' gestation and five patients have an ongoing pregnancy. This case series suggests the incidence of dizygotic/monochorionic triplets following IVF is approximately 10 times higher than the expected rate in unassisted conceptions, and underscores the importance of a conservative approach to lower the number of embryos at transfer. The role of embryo transfer technique and *in vitro *culture media in the twinning process requires further study.

## Background

With the advent of the advanced reproductive technologies, the rate of multiple gestation has increased. In Japan, the rate of monochorionic triplet pregnancy is reported to be 0.004% of natural pregnancies [1,2]. Although the rate of monochorionic triplet pregnancy is quite low, it has been reported that both monochorionic triplet pregnancy and monozygotic twins [3] is increased in the setting of IVF. In this report, we review features of seven unusual cases of multiple gestation established after IVF in Japan, and discuss some possible explanations for the observed findings.

## Case presentation

We reviewed records of all patients who become clinically pregnant via IVF at Denentoshi Ladies Clinic Reproductive Center between September 2000 and December 2006 (*n *= 2,433). In all cases, embryos were uniformly transferred using a Phycon IVF Catheter (Fuji System, Tokyo, Japan) under abdominal ultrasound guidance by the same individual. Seven cases of monzygous multiple gestation were identified, as described here (Table 1):

**Table 1:** Case presentation of Monozygotic multiple gestation

Case	Age (yrs)	History	Ovulation protocol	#ET	Culture	Embryo grade	Outcome (wks)
1	39	G2P1	GnRH-a Short HMG	1	5 days	5AA	9w
2	32	G0	GnRH-a Short HMG	2	5 days	4AA,4AB	33w4d
3	34	G1P0	GnRH-a long HMG	2	5 days	3AA,2AB	30w5d
4	32	G0	GnRH-a long HMG	2	3 days	8cellsG1,8cellsG1	37w2
5	30	G0	GnRH-a long HMG	2	5 days	4AA,3AA	40w3d
6	29	G0	CC+HMG+GnRH-ant	2	3 days	8cellsG1,7cellsG1	8w
7	40	G1P0	GnRH-a Short HMG	3	3 days	8cellsG1,8cellsG2	33w5d

#Number of Embryo Transfer

### Case 1

This Caucasian gravida 2 para 1 (age 37) presented with her partner for assessment and treatment of unexplained infertility of three years' duration. Her gynecological history was unremarkable. She had a regular 28 day menstrual cycle and all laboratory test results were normal. The couple underwent ovulation induction +IUI six times without success before opting for IVF. Following pituitary down-regulation with intranasal buserelin acetate (Buserecure, Fuji Pharmaceutical, Tokyo, Japan) initiated on day 2, controlled ovarian hyperstimulation was performed using a combined follicle stimulating hormone (FSH; Fertinom P, Serono, Tokyo, Japan) + human menopausal gonadotoropin (HMG; Humegon, Japan Organon, Osaka, Japan) protocol. Human chorionic gonadotropin 10,000 IU (hCG; Profasi, Serono, Tokyo, Japan) was administered 36 hours before retrieval. Ten oocytes were obtained via ultrasound-guided transvaginal needle aspiration, and were cultured in Universal IVF Medium (MediCult a/s; Jyllinge, Denmark) and BlastAssist System 1, 2 (MediCult a/s) in a 5% CO_2_, 5% O_2_, and 90% N_2 _environment. Eight oocytes were cultured for five days, and of these four developed to the blastocyst stage. The patient underwent a single blastocyst transfer (Gardner's classification: 5AA) on day 5 following ovum collection. The three non-transferred blastocysts (grade 5AA, 5AA and 4AA) were cryopreserved by vitrification (Cryotop and Vitrification Kit; Kitazato Supply, Shizuoka, Japan). Luteal progesterone support was given for two weeks. The patient achieved a pregnancy and delivered a healthy singleton infant.

Two years later, the patient returned for FET. One blastocyst (grade 5AA) was thawed using the Cryotop and the Vitrification Kit; and was transferred on day 5 with assisted hatching (AH) via acid Tyrode's method (ovulation occurred in a natural cycle). She achieved a successful pregnancy and a single gestational sac (GS) was observed at 5 weeks gestational age. Two weeks later, follow-up TV-USG confirmed a single gestational sac but three distinct amniotic sacs could now be visualized, each with distinct fetal poles and distinct cardiac activity. The risks of multiple gestation were carefully explained to the patient and the case was co-managed with a perinatologist. After extensive counseling, the couple decided to terminate the pregnancy at 9 weeks' gestation.

### Six additional cases (dizygotic triplet)

Case 2 (age 32). This patient sought IVF+ICSI for male factor infertility. Pituitary down-regulation was achieved with intranasal buserelin acetate (Buserecure, Fuji Pharmaceutical, Tokyo, Japan) initiated on day 2, controlled ovarian hyperstimulation was performed using a combined follicle stimulating hormone (FSH; Fertinom P, Serono, Tokyo, Japan) + human menopausal gonadotoropin (HMG; Humegon, Japan Organon, Osaka, Japan) protocol. Human chorionic gonadotropin 10,000 IU (Profasi, Serono, Tokyo, Japan) was administered 36 hours before retrieval. The patient requested transfer of two embryos following *in vitro *culture for five days with Quinn's Advantage medium (SAGE In-Vitro Fertilization. Inc., CT, USA). A positive pregnancy test was confirmed 14 days post-ET, and follow-up TV-USG revealed a dichorinoic-triamniotic triplet gestation. Cesarean delivery was performed at the 33^rd ^gestational week without complication.

Case 3 (age 34). This patient underwent IVF+ICSI for male factor infertility using the "long protocol" controlled ovarian hyperstimulation. Following culture for five days with Quinn's Advantage medium (SAGGE In-Vitro Fertilization. Inc., CT, USA), the patient requested transfer of two blastocysts. TV-USG study established the diagnosis of dichorinoic triamniotic triplet pregnancy, and an uncomplicated Cesarean delivery was performed at 30 weeks' gestation.

Case 4. This patient (age 32) underwent IVF+ICSI for male factor infertility using the "long protocol" controlled ovarian hyperstimulation. After a three-day incubation with Quinn's Advantage medium (SAGE In-Vitro Fertilization. Inc., CT, USA), two embryos were transferred. At 8 weeks' gestation, the diagnosis of dichorinoic-triamniotic triplets was made via TV-USG. While this case resulted in the spontaneous loss of the monozygotic twins by 9 weeks' gestation, the surviving (singleton) gestational sac continued to 37 weeks' gestation and resulted in the vaginal delivery of a healthy infant.

Case 5. This patient (age 30) underwent IVF+ICSI for combined female (tubal) and male factor infertility. Ovarian stimulation (mixed FSH+hMG protocol) followed pituitary downregulation with gonadotropin-releasing hormone agonist (Buserecure; Fuji Pharmaceutical, Tokyo, Japan) starting from the midluteal phase of the preceding cycle. Twelve oocytes were retrieved; the embryos were cultured with Quinn's Advantage medium (SAGE In-Vitro Fertilization. Inc., CT, USA). Two blastocysts (grade 4AA and 3AA) were transferred on day five. Dichorinoic-triamniotic triplets were diagnosed via TV-USG. at 8 weeks' gestation. Within a week, the monozygotic twin set was lost by spontaneous abortion. The singleton pregnancy continued to term (40 weeks) and resulted in a normal vaginal delivery.

Case 6. This patient (age 29) presented with her partner for IVF+ICSI with a diagnosis of male factor infertility. The patient received a mixed stimulation protocol consisting of clomiphene citrate and recombinant FSH. GnRH antagonist was used to prevent endogenous LH signaling. Oocytes were cultured in Universal IVF Medium (MediCult a/s; Jyllinge, Denmark) and BlastAssist System 1, 2 (MediCult a/s) in a 5% CO_2_, 5% O_2_, and 90% N_2 _environment. After a two-embryo transfer, a dichorinoic-triamniotic triplet pregnancy was diagnosed via TV-USG. Risks associated with multiple gestation were explained to the patient in detail, and a second opinion was provided by a perinatologist. After extensive counseling, the couple decided to terminate the pregnancy at 8 weeks' gestation.

Case 7. This patient (age 40) presented with her partner for assessment and treatment of tubal factor infertility, having already undergone five unsuccessful IVF cycles elsewhere. The "short protocol" for controlled ovarian hyperstimulation was chosen. Three oocytes were collected and all advanced to the 2*pn *stage after conventional fertilization. Embryos were cultured for three days with Quinn's Advantage medium (SAGE In-Vitro Fertilization. Inc., CT, USA) and the patient requested transfer of all three. One 8-cell [Veeck]G1, and two 8-cell [Veeck]G2 embryos were transferred on day 3. TV-USG performed at 8 weeks' gestation demonstrated two gestational sacs: two fetal poles in one sac and one fetal pole in the other sac. Each fetus was associated with an independent amniotic membrane, consistent with dichorinoic-triamniotic triplet. A cerclage was placed at 12 weeks' gestation. The patient was hospitalized at 33 weeks due to premature rupture of membranes, and a cesarean section was performed at 33 5/7 weeks' gestation.

## Discussion

Multiple gestation is associated with well known increases in obstetrical risk; moreover, the frequency of multiple gestation increases in IVF where more than one embryo is transferred. Monochorionic triplet pregnancy represents a very rare subset of multiple gestation, and occurs only in approximately 0.004% of pregnancies [1,2]. Interestingly, some researchers have reported an incidence of monochorionic triplet pregnancy after IVF approximately 100 times higher than that occurring in natural (unassisted) pregnancy [4,5]. At our center the incidence of monochorionic triplet pregnancy was calculated at 0.048%, some 10 times higher than the rate observed in natural pregnancy. Since this ratio was calculated from clinical pregnancy data (and the successful pregnancy outcome rate was unknown), the actual ratio is likely to be higher. Although the literature contains a number of reports describing this phenomenon [6,7], the exact mechanism whereby IVF influences the frequency of monochorionic triplet pregnancy remains controversial.

Several factors have been offered to explain the mechanism whereby the advanced reproductive technologies increase the rate of monochorionic pregnancy. These include ovulation induction [8], *in vitro *culture conditions [9], micromanipulation of the zona pellucida [10], and patient history [11]. Assisted embryo hatching (AH) and ICSI have also been implicated as factors in the monochorionic equation [12].

Our data do not find AH and ICSI making an important contribution to the incidence of triplets, a finding consistent with data on monozygous twins in IVF reported from the Cornell group [13]. Henne et al suggested that patients who undergo AH with ICSI have an *a priori *poor prognosis due to advanced age [7]. Similarly, Elizur *et al *concluded that monozygotic twinning is not associated with zona pellucida micromanipulation procedures [14]. It has been suggested that some IVF patients may have a degree of cytoplasmic fragility and that the incidence of triplets does not increase for an independent reason other than manipulation of the zona pelucida [3]. It has been suggested that ovarian stimulation itself increases the incidence of multiple gestation. It should be noted that monochorionic triplet increases following ovulation induction, and that gonadotropin exposure during ART is one of the reasons [15]. Moreover, it is suspected that prolonged embryo culture and associated blastocyst transfer may increase the incidence of monochorionic triplet [9]. Yet many clinics now perform blastocyst transfer and it seems unlikely that this is an important reason for the increase in incidence of multiple gestation following IVF. According to one report [15], the mechanism leading to a monochorionic multiple gestation may be related to a weakening of the cell before implantation and a separation of the inner cell mass via an unknown process. The cases presented here do not shed light on this unknown mechanism. As multiple gestation increases pregnancy complications [1] and perinatal mortality [16,17], efforts to reduce such outcomes are welcome. At present, an increased risk for multiple gestation exists whenever multiple embryos are transferred with IVF. However, further study of the embryo culture microenvironment, method of ovarian stimulation process, and the approach to embryo transfer (*i.e*., single embryo transfer) [18] may help lower the incidence of monochorionic triplet pregnancy to the natural rate.

## Authors' contributions

AY was the primary author of the manuscript and carried out statistical evaluation. TY, HM, TK were involved in management of the IVF. HW was mainly culture the embryo. All authors read and approved the final manuscript.
